# Paraproteinemic keratopathy in monoclonal gammopathy of undetermined significance treated with primary keratoprosthesis: Case report, histopathologic findings, and world literature review

**DOI:** 10.1097/MD.0000000000008649

**Published:** 2017-12-15

**Authors:** Homer H. Chiang, Rebekah S. Wieland, Thomas S. Rogers, Pamela C. Gibson, George Atweh, Gregory McCormick

**Affiliations:** a University of Vermont College of Medicine; b Department of Pathology and Laboratory Medicine; c Department of Hematology and Oncology, University of Vermont Medical Center; d Ophthalmic Consultants of Vermont, South Burlington, VT.

**Keywords:** crystalline, keratopathy, keratoprosthesis, monoclonal gammopathy of undetermined significance

## Abstract

**Rationale:**

We report a case of paraproteinemic keratopathy associated with monoclonal gammopathy of undetermined significance, treated with keratoprosthesis as a primary penetrating procedure. Histopathological findings and a world literature review are presented.

**Patient concerns:**

A 74 year old female recently diagnosed with monoclonal gammopathy undetermined significance presented with progressive blurry vision bilaterally.

**Diagnoses:**

Examination revealed corneal opacities consistent with paraproteinemic keratopathy.

**Interventions:**

Corneal transplantation with the Boston Type I keratoprosthesis was performed on the right and, a year later, on the left.

**Outcomes:**

Visual outcomes were good. Histopathological staining of host corneal buttons were consistent with monoclonality, and electron microscopy revealed fibrillar extracellular aggregates within intervening normal stroma.

**Lessons:**

Corneal deposits may be the only manifestation of monoclonal gammopathy of undetermined significance in patients who are otherwise systemically asymptomatic. Ophthalmologists who encounter corneal opacities may order the appropriate diagnostic studies to determine the presence of occult systemic disease. Risk of graft failure after penetrating keratoplasty from recurring opacities is high, so keratoprosthesis as a primary penetrating procedure may afford superior long-term outcomes. Host corneal buttons retrieved from penetrating keratoplasty or corneal biopsy may be sent for histopathological examination to confirm the diagnosis.

## 1 Introduction

Monoclonal gammopathy of undetermined significance (MGUS) is the most common lymphoplasmacytic proliferative disorder and a potential premalignant clonal plasma cell condition. It is defined by the presence of a serum monoclonal protein at a concentration less than 3 g/dL, a bone marrow with less than 10% monoclonal plasma cells and absence of end organ damage including lytic bone lesions, anemia, hypercalcemia, renal insufficiency, hyperviscosity.^[1]^ Typically, patients with MGUS remain asymptomatic and stable for many years with close follow-up. However, a large proportion do progress to premalignant and malignant monoclonal gammopathies such as smouldering multiple myeloma, multiple myeloma, Waldenström's macroglobinemia, primary amyloidosis, B-cell lymphoma, or chronic lymphocytic leukemia.^[2]^

Corneal deposits have long been associated with monoclonal gammopathies since first described by Meesman in 1934,^[3]^ and is estimated to occur in 1% of cases.^[4]^ Once thought to be lipid deposits, Klintworth et al^[5]^ in 1978 determined that many were indeed “crystalline” deposits associated with paraproteinemias. Here, we provide a world literature review of reports of corneal deposits associated with paraproteinemias following Garibaldi et al review in 2005.

## 2 Case report

A 74-year-old Caucasian female presented for corneal consultation complaining of 8 years of progressive blurry vision in both eyes. Her past medical history was significant for hyperlipidemia and a recent diagnosis of MGUS based on an elevated IgG and free lambda light chain level. Her ocular history included cataract extraction with posterior chamber intraocular lens implantation, a diagnosis of presumed “lipid keratopathy” in both eyes, and progressive irregular astigmatism treated with a rigid contact lens. Visual acuity with rigid gas permeable contact lenses (RGPCL) was 20/70 in the right and 20/20 in the left.

**Figure 1:**
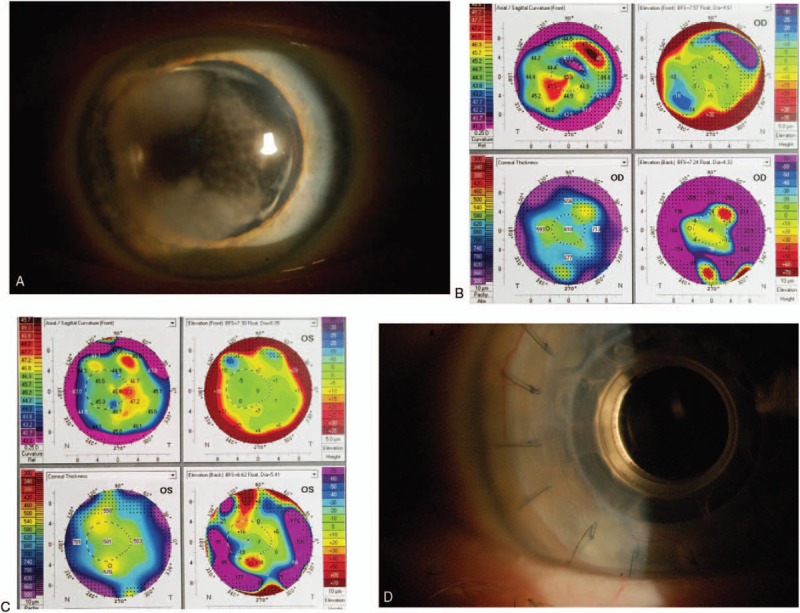
Slit lamp color photographs of right eye (A). Corneal topography of the right (B) and left (C) eye. (D) Slit lamp color photograph demonstrating recurrence of corneal opacity in the donor cornea 1 year after keratoprosthesis implantation.

Examination revealed fluffy white corneal opacities in the all layers of the stroma, in an annular distribution spanning 9 clock hours, bilaterally (Fig. 1A). The opaque corneal deposits were associated with thickened stroma and associated corneal surface elevation. A 1.5 to 3 mm lucent area was present between the deposit and the limbus. The central cornea was affected to a lesser degree but limited best corrected visual acuity (BCVA) in the right eye. Ultrasound pachymetry revealed increased central corneal thickness bilaterally, and topography revealed irregular astigmatism of both eyes with inferotemporal steepening in the right and temporal steepening in the left (Fig. 1B and C). The remainder of the examination, including the posterior segment, was unremarkable. The patient reported no symptoms of systemic disease.

Differential diagnosis includes macular and Schnyder corneal dystrophy, deposits secondary to congenital metabolic disorders, and lipid deposits secondary to lipoproteinemia. In the setting of MGUS and the above-noted clinical findings, a diagnosis of paraproteinemic crystalline keratopathy was made and treated with observation. Over the next year, the patient's BCVA with RGPCL continued to worsen as the stromal deposits progressed in the visual axis bilaterally to 20/150 in the right eye and 20/50 in the left eye. Treatment options, including continued observation, penetrating keratoplasty, and Boston type 1 keratoprosthesis, were considered. After discussing the risk of recurrence of stromal deposits in traditional penetrating keratoplasty, the patient elected primary keratoprosthesis implantation in the right eye. Postoperative BCVA in the right eye was 20/25. A year later, vision in the left eye worsened to 20/200 due to progression of stromal opacity, and a Boston type 1 keratoprosthesis was implanted into the left eye, with a postoperative BCVA of 20/20. The host corneal buttons obtained were sent for histopathologic examination and electron microscopy.

At 1 year follow-up in the right eye, the patient's BCVA was maintained at 20/25. Corneal opacity suggestive of recurrent paraproteinemic keratopathy developed in the donor cornea in the right eye, but central vision through the optic cylinder was preserved (Fig. 1D).

## 3 Histological description of corneal deposits

**Figure 2:**
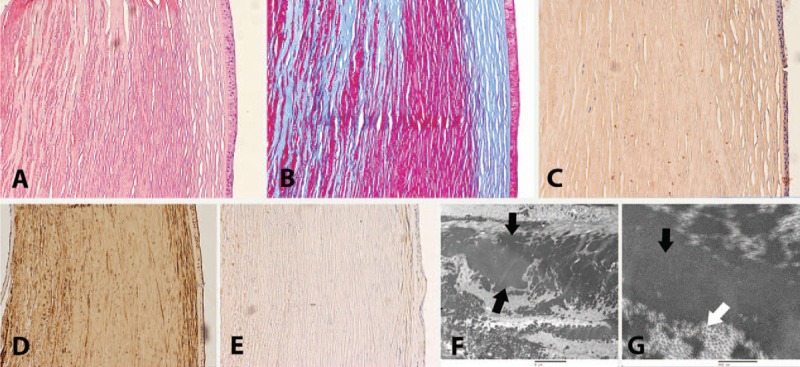
Hematoxylin and eosin (A) stain of cornea from the right eye at ×100 magnification highlights abnormal hypereosinophilic deposits within the cornea's normal stroma. Trichrome stain (B) highlights abnormal red deposits in the normal stromal collagen staining blue. Congo red stain (C) lacks the salmon pink staining typically seen in amyloid deposition (negative for amyloid). Lambda stain (D) shows positive brown staining of the corneal stroma compared with the Kappa stain (E) which is negative; electron microscopy cornea from right eye demonstrates fibrillary deposits between the black arrows on (F) 2 μm view, with normal stromal collagen of the cornea indicated by the white arrow on (G) 500 nm view.

Histologically, both the left and right corneas showed proteinaceous appearing deposits in the corneal stroma forming a diffuse to nodular thickening of the corneal stroma, which were hypereosinophilic on hematoxylin and eosin stain (Fig. 2A) and fuchsinophilic on trichrome stain (Fig. 2B). The deposits were Periodic acid-Schiff. positive and Congo red stain negative, suggesting this did not represent amyloid deposition (Fig. 2C). Immunohistochemical stains showed strong staining for lambda (polyclonal, Dako) but not kappa (polyclonal, Dako) supporting the material represented monoclonal paraprotein (Fig. 2D and E). The material did not have a crystalline appearance on light microscopy.

Portions of the right cornea were also submitted for electron microscopy. The initial thin tissue sections were stained with toluidine blue, revealing well-oriented portions of relatively normal-appearing cornea as well as portions of cornea with dark deposits within the stroma. On ultrastructural analysis, the epithelial cells were relatively unremarkable. The majority of the Bowman layer appeared unremarkable with rare areas in which deposits were present. Within the corneal stroma extracellular fibrillar deposits were arranged in short parallel and occasionally arcing aggregates. The fibrils had an electron dense core surrounded by a corona of lighter density (Fig. 2F and G). The central core averaged 6.7 nm in diameter with a range of 4.3 to 10.10 nm. The fibrils were intermixed with normal stromal collagen.

## 4 Discussion

MGUS keratopathy has no clear medical treatments. Patients may be asymptomatic and may be observed with regular follow-up. Visual symptoms including decreased vision and photophobia may occur secondary to corneal opacification or irregular increases in corneal thickness resulting in irregular astigmatism. On slit lamp examination, patients may demonstrate deposits in all the layers of the corneal stroma. The appearance of the deposits varies by report, ranging from cloudy and amorphous to a more crystalline appearance. The pattern of deposits ranges from diffuse, extending to the periphery, to more annular and central as in this case.

The etiology of corneal immunoprotein deposits is currently unclear. Multiple mechanisms have been postulated including deposition via the limbal vasculature, tear film, aqueous, and production by keratocytes. These hypotheses are based on variations in location of deposits anteroposteriorly within the stroma and coronally within the cornea (i.e., central and perilimbal clear zones). These mechanisms correspond to the progressive nature of the deposits. In the majority of cases, visual acuity worsened over time on the order of years. In several cases, including our patient, deposits were initially in an annular distribution with clear central and perilimbal zones, and over time progress centrally to involve the visual axis.

With the exception of intracellular production, recurrence of deposits in the donor cornea following penetrating keratoplasty is plausible with each of these mechanisms. In our literature review (Table 1 ), since 2005 four patients experienced recurrence of deposits following penetrating keratoplasty, with 1 patient experiencing recurrent deposits following 2 consecutive penetrating keratoplasties. Two patients experienced recurrence following superficial keratectomy. In 3 of the 4 cases with recurrence following penetrating keratoplasty, deposits were present in all the layers of the stroma. The precise location of deposits was not described in the fourth case.

**Table 1:**
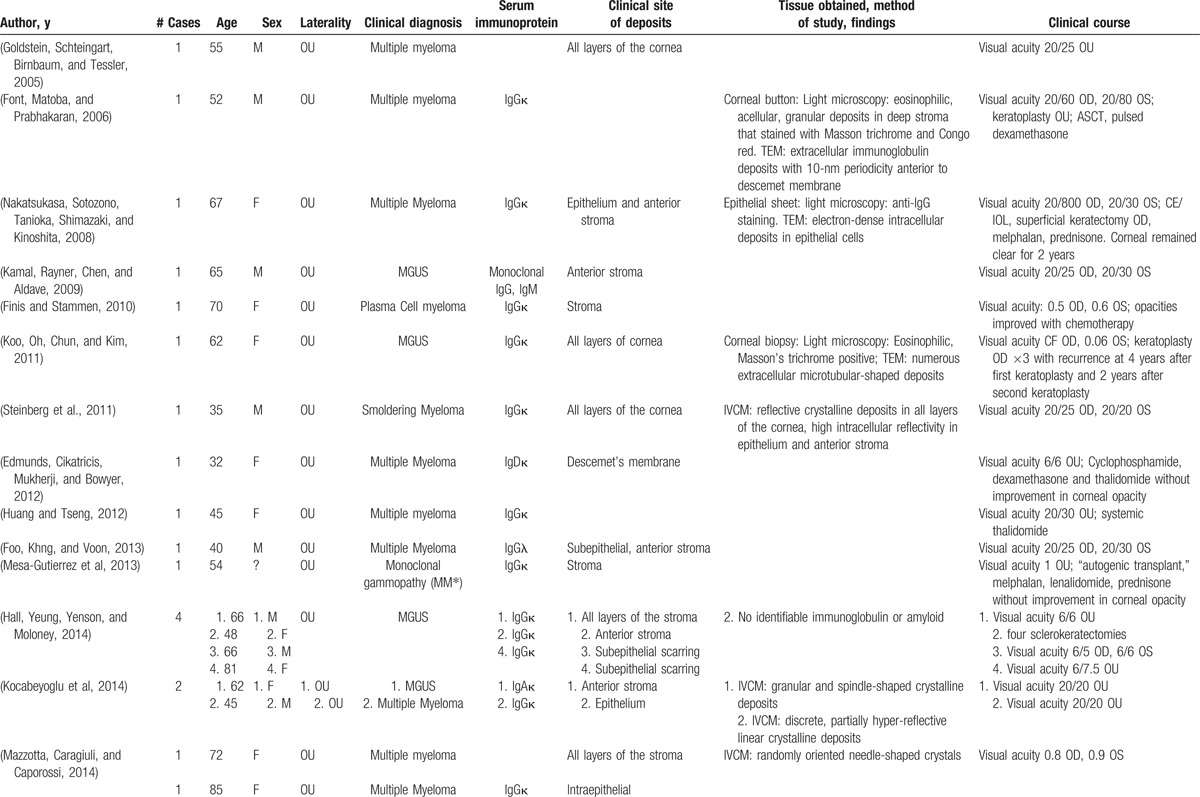
Reports of immunoprotein corneal deposits in patients with systemic disease.

**Table 1 (Continued):**
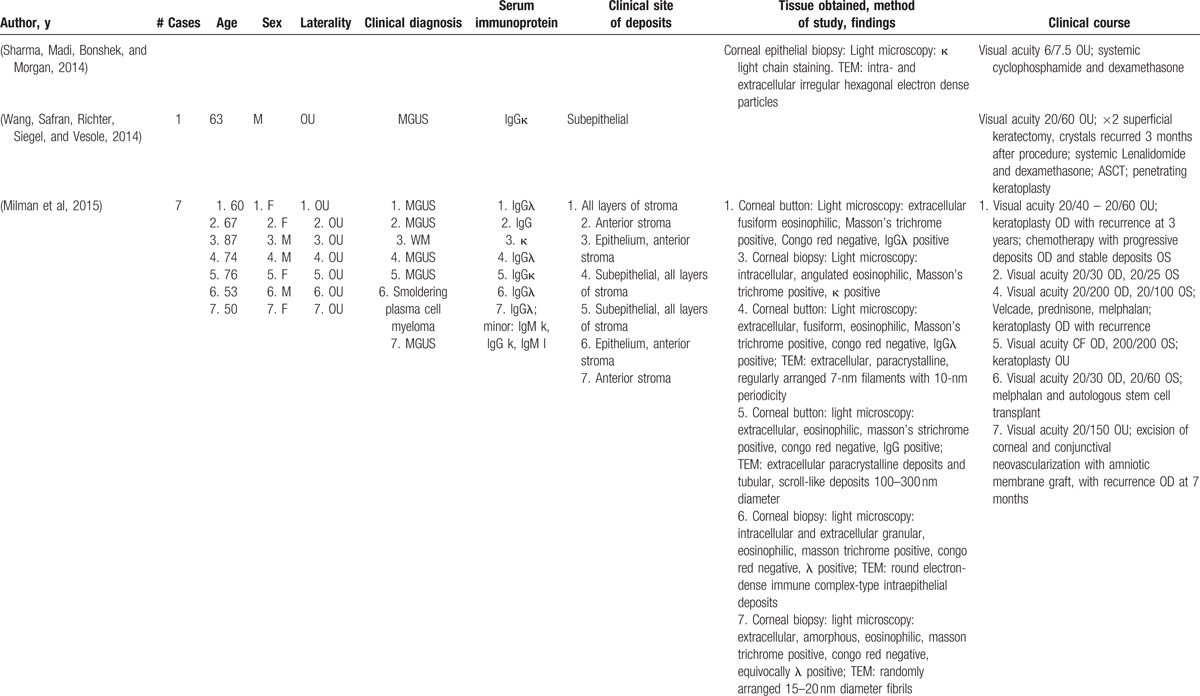
Reports of immunoprotein corneal deposits in patients with systemic disease.

In symptomatic patients with corneal opacity associated with multiple myelomea, some case reports suggest visual improvement with systemic plasma exchange and chemotherapy.^[3]^ However, corneal deposits may clinically progress despite serological improvement of serum immunoglobulin levels.^[6]^ Our patient showed progressive corneal opacity despite optimal systemic management of MGUS. Limited evidence has not shown steroid eyedrops to be effective in halting or reducing the density of deposits perhaps in part because the deposits are not associated with stromal vascularization. Patients with MGUS associated corneal opacity treated with penetrating keratoplasty have been shown to develop visually significant, recurrent corneal opacity after a period ranging from 1 month to 3 years after surgery.^[6,7]^Thus patients with MGUS-associated corneal opacity are at high risk of corneal graft failure due to recurrent corneal opacification and may have superior long-term outcomes with keratoprosthesis implantation as a primary penetrating procedure. Increasing evidence has demonstrated primary keratoprosthesis as a safe and effective option for conditions in which penetrating keratoplasty carries a poor prognosis.^[8]^ The risks associated with keratoprosthesis may be preferable to the problems associated with repeat penetrating keratoplasty, including allosensitization leading to increased risk of graft rejection and failure.^[9]^

Limitations of this report include generalizability based on keratoprosthesis availability, patient selection, and rarity of disease. While keratoprosthesis is being increasingly utilized, it is not offered at most centers. It is still widely considered to be a second-line treatment modality after traditional keratoplasty has failed. The most common complications associated with keratoprosthesis—retroprosthetic membrane, elevated intraocular pressure, and vitritis/endophthalmitis—require close collaboration between cornea, glaucoma, and retina specialists familiar with the device. Further, patients who are candidates for keratoprothesis must be motivated to adhere to the strict regimen of topical medications, bandage contact lens care, and regular follow-up. Finally, corneal deposits are a rare manifestation of MGUS, so larger studies of the efficacy of keratoprosthesis will be challenging.

## 5 Conclusion

While a rare manifestation, corneal deposits may signify the presence of otherwise clinically silent paraproteinemias and other hematologic dyscrasias. For patients in whom these diagnoses are considered, the ophthalmologist may investigate with total serum protein, serum protein electrophoresis, serum free light chains, bone marrow biopsy and aspirate, and flow cytometry. Corneal specimens obtained from biopsy or penetrating keratoplasty may be sent for pathologic analysis with immunohistochemical staining and electron microscopy. There is currently no consensus on the medical management of corneal deposits secondary to MGUS, and corneal opacification may continue to progress despite serological improvement. Patients treated surgically with penetrating keratoplasty face a high risk of recurrence making primary keratoprosthesis an appropriate consideration with the potential for superior long-term visual improvement.
